# Data exploration on diet, and composition, energy value and functional division of prey items ingested by White Storks *Ciconia ciconia* in south-western Poland: Dietary variation due to land cover, reproductive output and colonial breeding

**DOI:** 10.1016/j.dib.2018.10.064

**Published:** 2018-10-24

**Authors:** Grzegorz Orłowski, Jerzy Karg, Leszek Jerzak, Marcin Bocheński, Piotr Profus, Zofia Książkiewicz-Parulska, Karol Zub, Anna Ekner-Grzyb, Joanna Czarnecka

**Affiliations:** a Institute of Agricultural and Forest Environment, Polish Academy of Sciences, Bukowska 19, 60-809 Poznań, Poland; b Department of Nature Conservation, Faculty of Biological Sciences, University of Zielona Góra, Zielona Góra, Poland; c Institute of Nature Conservation, Polish Academy of Sciences, Kraków, Poland; d Department of General Zoology, Faculty of Biology, Adam Mickiewicz University, Poznań, Poland; e Mammal Research Institute, Polish Academy of Sciences, Bialowieża, Poland; f Department of Behavioural Ecology, Faculty of Biology, Poznań, Poland; g Department of Ecology, Institute of Biology and Biochemistry, Maria Curie-Skłodowska University in Lublin Poland

## Abstract

The dataset presented in this data paper supports "Linking land cover satellite data with dietary variation and reproductive output in an opportunistic forager: Arable land use can boost an ontogenetic trophic bottleneck in the White Stork *Ciconia* ciconia" (Orłowski et al. 2019) [1]. Analysis of data on diet and prey composition based on an investigation of 165 pellets of White Storks *Ciconia ciconia* sampled from 52 nests showed that their diet was based primarily on ‘eurytopic prey’ (embracing taxa from grassland and a variety of non-cropped habitats), the biomass contribution of which in the diet was disproportionately (3–4–fold) higher than the percentage of available corresponding habitats. Similarly, prey items from water/wetland sites prevailed over the availability of corresponding habitats. The opposite pattern characterized prey taxa from arable habitats and forests, the contribution of which was lower than the availability of the corresponding habitats. The total energy content per pellet (calculated by summing the energy content of all individual prey items across one specific prey group) was the most strongly correlated with the biomass of Orthoptera, thereafter with that of mammals, other vertebrates, earthworms and other invertebrates, but not with the biomass of Coleoptera. White Storks from nests of low productivity pairs (i.e. with 1–2 fledglings) consumed a significantly (up to two-fold) higher biomass of Coleoptera, Orthoptera and all invertebrates, which also translated into a higher total biomass and a higher total energy content compared to the diet of high-productivity pairs (i.e. with 3–4 fledglings). Our data, in particular those relating to energy content in a variety of invertebrate taxa, and their body mass and functional division in terms of habitat preferences should be useful for other researchers to calculate energy budgets of predatory animals living in agricultural landscapes in Europe.

**Specifications table**

**Table:** 

Subject area	Ecology, Biological Sciences
More specific subject area	Foraging and Dietary Ecology
Type of data	Tables and Figures
How data was acquired	Through field work and laboratory work
Data format	Raw, filtered and analysed
Experimental factors	Investigation of 165 pellets of White Storks *Ciconia ciconia* sampled from 52 nests in June and July 2012 in 39 villages in south-western Poland.
Experimental features	The identification of each prey items consumed along with their dry weights and eco-morphological characteristics: energy content (expressed in kJ) and functional division in terms of habitat preferences.
Data source location	Turew, SW Poland, Research Station of Institute of Agricultural and Forest Environment, Polish Academy of Sciences
Data accessibility	The data are given in this article
Related research article	G. Orłowski, J. Karg, L. Jerzak, M. Bocheński, P. Profus, Z. Książkiewicz-Parulska, K. Zub, A. Ekner-Grzyb, J. Czarnecka, Linking land cover satellite data with dietary variation and reproductive output in an opportunistic forager: Arable land use can boost an ontogenetic trophic bottleneck in the White Stork *Ciconia ciconia*. Sci. Tot. Environ. 646 (2019) 491–502.

**Value of the data**

• The data presents the functional classification of prey items into four major habitat categories: (i) arable, (ii) grassland/non-cropped (= marginal habitats = eurytopic prey), iii) forest; and iv) water/wetland and could be used by others researchers.
• For each prey taxa the data on energy content based on ash-free dry mass is given and this data could be re-used in other studies.
• The data in this article, in particular those on energy content in a variety of invertebrate taxa, and their body mass and functional division in terms of habitat preferences, should be useful for other researchers to calculate energy budgets of predatory animals.

## 1 Data

**Fig. 1:**
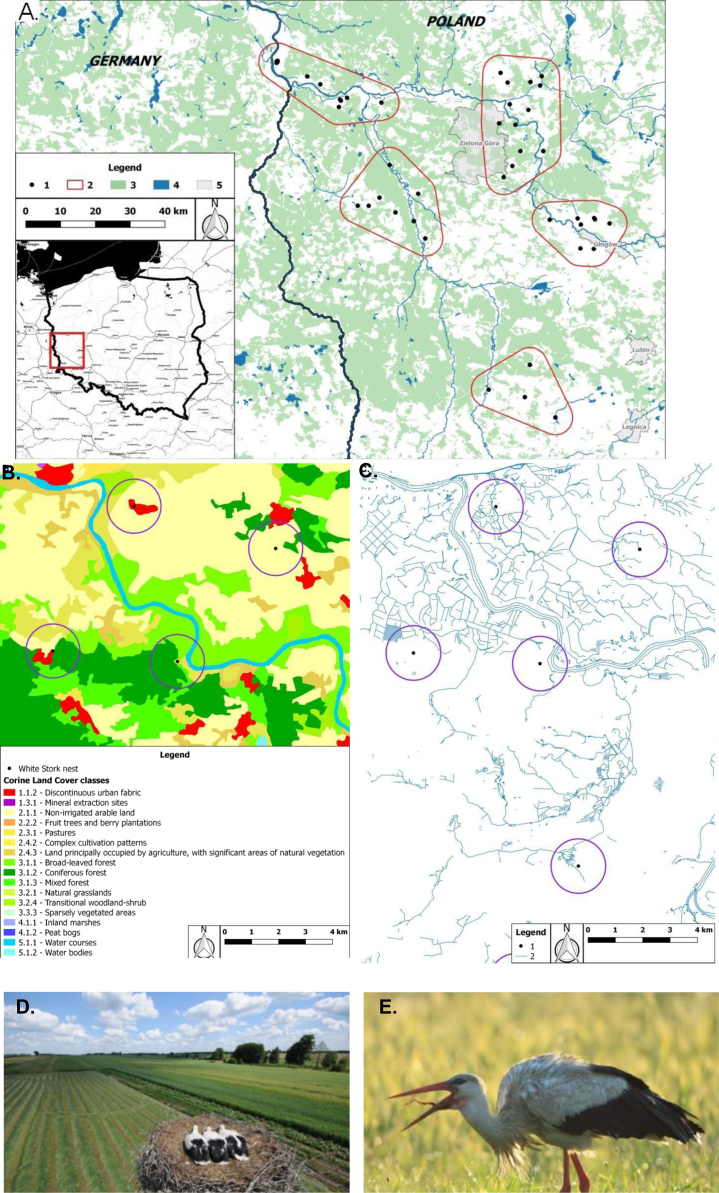
(A) General map showing the distribution of 52 nests (black dots) of White Storks clustered within five sub-plots (between 373 and 764 km^2^ in area) in south-western Poland where pellets were sampled for dietary analysis; (2) border of five subplots, (3) forest, (4) water/hydrographic network, (5) other land cover types. (B) The land cover types representing the class 3 of the Corine Land Cover classification. (C) The hydrographic networks around the sub-sample of nests, a circle of 1 km radius. (D) Land use around a nest of a high-productivity pair with three fledglings at the time the young were ringed (Photo credit: Adam Dmoch/www.birdwatching.pl). (E) An adult foraging on earthworms (Photo credit: Marcin Lenart/www.birdwatching.pl).

**Fig. 2:**
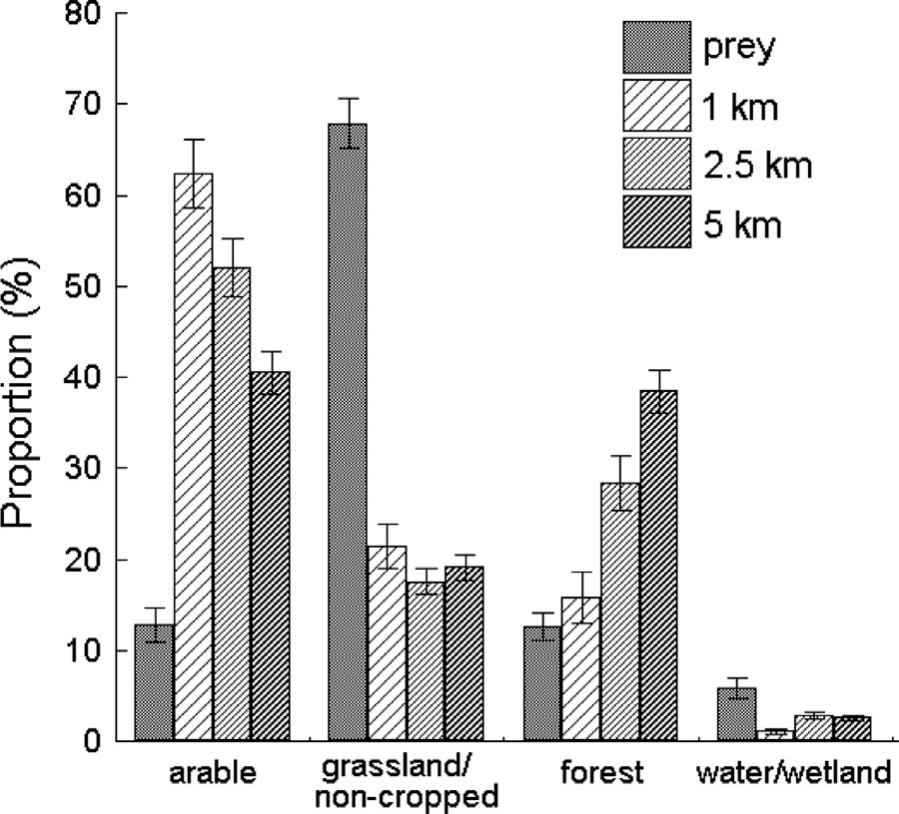
Comparison of the percentage distribution (average ± 1 SE per nest) of prey biomass (*n* = 20 561 items from 165 pellets) consumed by breeding White Storks, representing taxa classified into four major habitat categories: (i) arable, (ii) grassland/non-cropped (= marginal habitats = eurytopic prey), iii) forest; and iv) water/wetland (see Table 2) against the corresponding distribution of available landscape/habitat traits within three distances (1 km, 2.5 km and 5 km) determined for the same 52 nests of the species in south-western Poland. The landscape/habitat trait pools the following land cover classes (for land cover codes see Table 1): arable (ARA + HET), grassland/non-cropped (URB + IND + MIN + GRA + SHR), forest (FOR + ART) and water/wetland (WET + WAT + large rivers). Note that the *t*-test for dependent samples comparing the percentage distribution of an individual prey group vs landscape/habitat traits at successive distances for the same nests showed significant differences for most paired comparisons (*P* ≤ 0.011); the only non-significant comparison was for the prey/habitat category ‘forest’ at the distance of 1 km (*P* = 0.305).

**Fig. 3:**
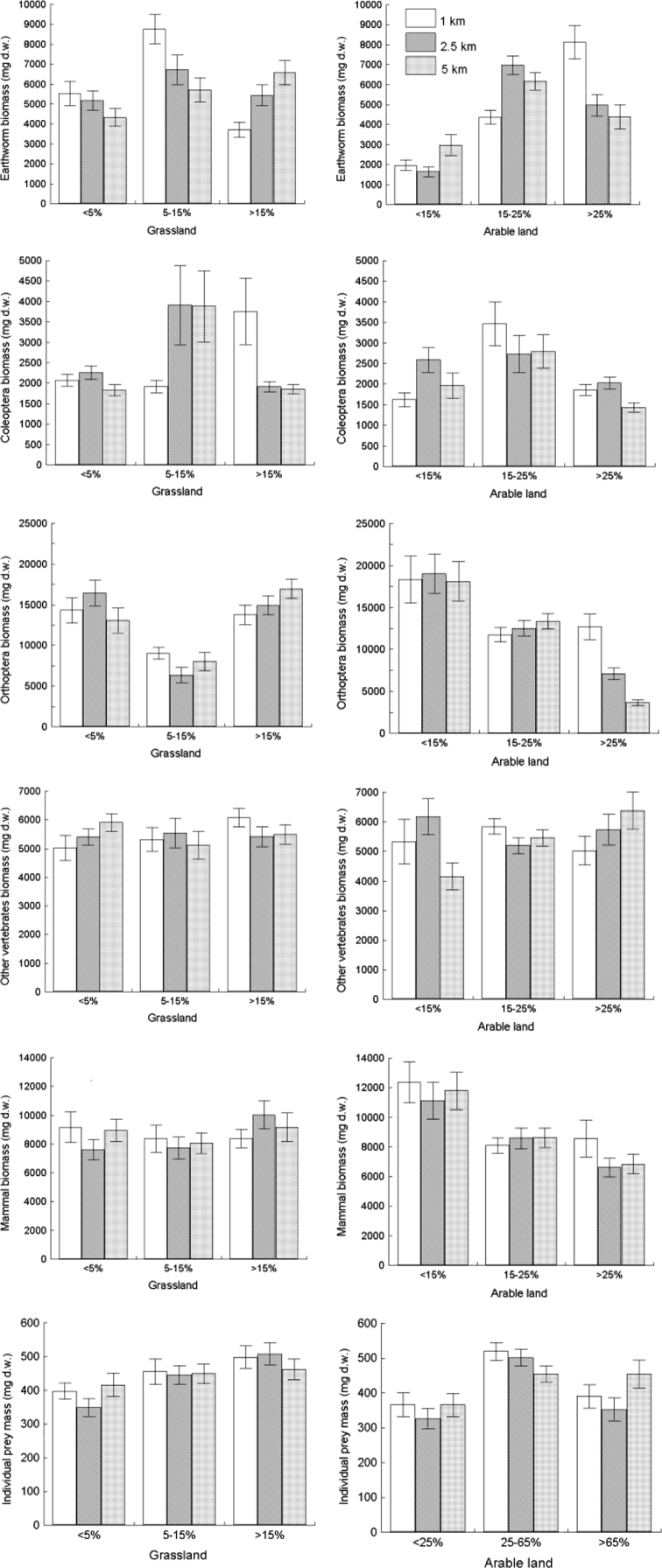
Biomasses of five major prey groups (earthworms; Coleoptera; Orthoptera; other vertebrates; and mammals) and individual prey mass per pellet (*n* = 165) compared for three spatial scales (extent/radius: 1 km, 2.5 km and 5 km) around White Stork nests and varying in percentages of grassland and arable land.

**Fig. 4:**
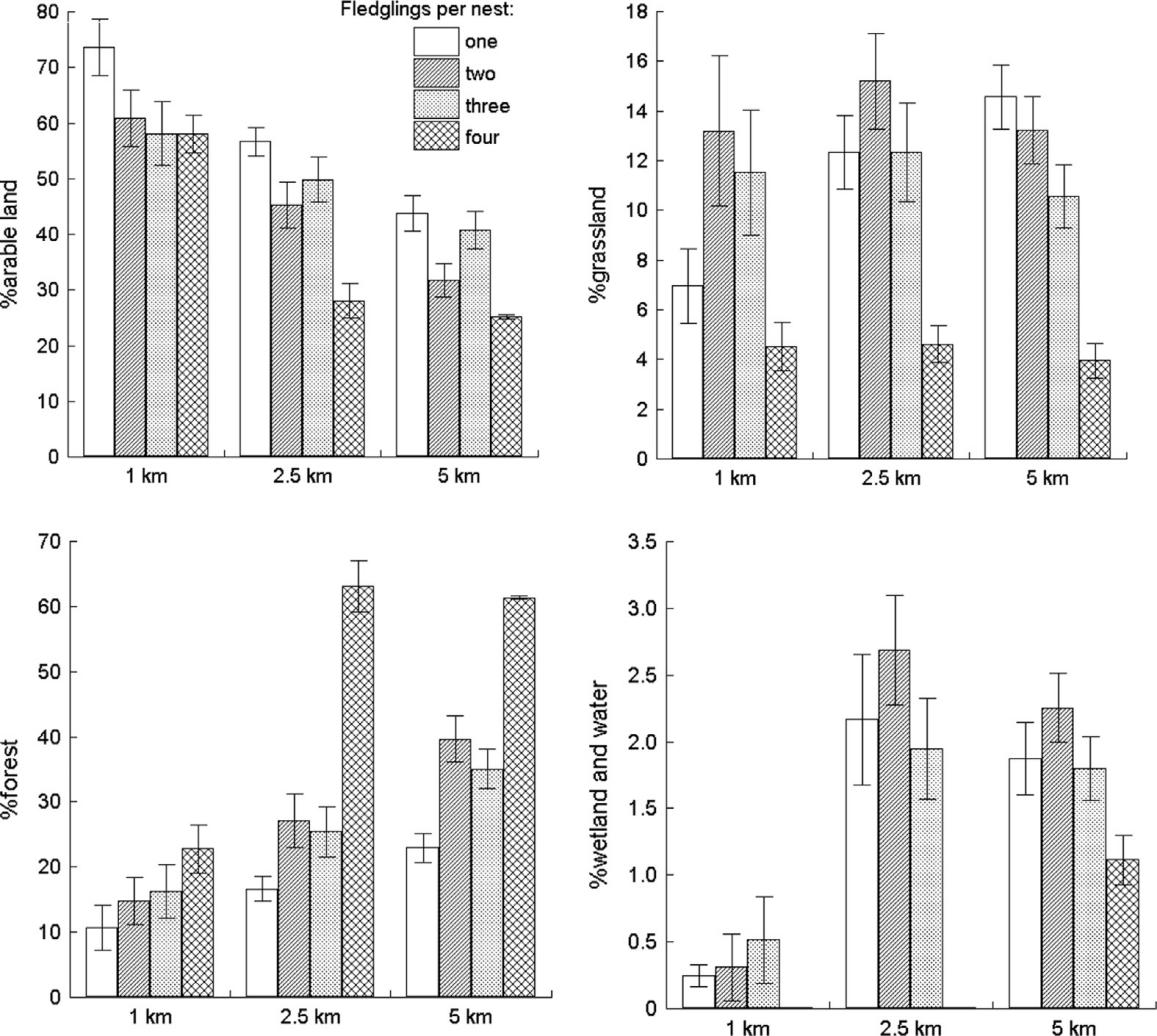
Comparison of the percentage variation of four land cover types (arable, grassland, forest and wetlands/water) measured within three distances around White Stork nests with different numbers of fledglings.

The data presented here were the basis for the article by Orłowski et al. [1]. The dataset of this article provides detailed information on dietary composition of 165 pellets of White Storks collected in June and July 2012 from 52 nests in 39 villages in south-western Poland (Fig. 1, Fig. 2, Fig. 3, Fig. 4, Table 1, Table 2, Table 3, Table 4, Table 5). The data describes basic dietary indices relating to prey items consumed, including the biomass contribution of invertebrate and vertebrate prey (Fig. 2, Fig. 3, Table 1, Table 2, Table 3, Table 4, Table 5).

**Table 1:** Major land cover types (class 2 of the Corine Land Cover classification) and traits of hydrographic networks determined for three radii (1 km, 2.5 km and 5 km) around 52 White Stork nests in south-western Poland. More information on the more detailed land cover types representing the class 3 of the CLC classification incorporated into the present class 2 of the CLC classification can be obtained on request from the authors. In the five subplots (2614 km^2^ in total; see Fig. 1A) the overall percentages of the major land cover types were: Urban fabric (4.3%); Industrial, commercial and transport units (0.8%); Mine, dump and construction sites (0.3%); Artificial non-agricultural vegetated areas (0.3%); Arable land (38%); Grassland, pasture (7.1%); Heterogeneous agricultural areas (3.1%); Forests (43.4%); Shrub and/or herbaceous vegetation associations (1.2%); Inland wetlands (2.8%); Inland waters (1.2%).

Land cover type, hydrographic trait	Class 2 of Corine Land Cover classification	Distance around nests (total area within a given distance)		
		1 km (314.1 ha)	2.5 km (1931.4 ha)	5 km (7725.4 ha)
Urban fabric (ha)	1.1	27.90 (±3.47)	67.53 (±10.41)	337.64 (±27.91)
Industrial, commercial and transport units (ha)	1.2	0	3.90 (±1.88)	123.02 (±21.92)
Mine, dump and construction sites (ha)	1.3	2.09 (±1.71)	6.74 (±3.70)	41.64 (±7.63)
Artificial non-agricultural vegetated areas (ha)	1.4	0.15 (±0.15)	14.25 (±3.17)	77.92 (±16.47)
Arable land (ha)	2.1	188.3 (±12.2)	915.4 (±57.7)	2849.9 (±184.1)
Grassland, pasture (ha)	2.3	35.9 (±6.0)	248.9 (±27.0)	875.7 (±74.3)
Heterogeneous agricultural areas (ha)	2.4	7.57 (±2.30)	88.24 (±9.06)	280.33 (±17.57)
Forests (ha)	3.1	49.6 (±8.8)	534.0 (±59.3)	2894.6 (±187.6)
Shrub and/or herbaceous vegetation associations (ha)	3.2	1.34 (±0.75)	11.74 (±3.44)	96.19 (±11.43)
Inland wetlands (ha)	4.1	0	0.45 (±0.33)	10.23 (±4.50)
Inland waters (ha)	5.1	1.25 (±0.64)	40.17 (±5.60)	138.25 (±14.18)
Watercourses (m)	–	9859.6 (±748.7)	52,118.6 (±3119.9)	164,602.7 (±7337.6)
Shoreline of water bodies (m)	–	1994.4 (±336.3)	16,103.7 (±1547.4)	57,922.6 (±4312.9)
Inland waters and large rivers (ha)	–	3.40 (±0.92)	53.68 (±6.59)	199.76 (±20.15)

**Table 2:** List of all the prey items (*n* = 20,561) representing six major prey groups (earthworms; Orthoptera; Coleoptera; other invertebrates; fish, reptiles and birds = other vertebrates; and mammals) taken by breeding White Storks *Ciconia ciconia* and identified in 165 pellets sampled in south-western Poland in 2012; individual dry masses of insects and certain invertebrates after and Karg, unpubl; estimate of earthworms consumed from [5]. The habitat preferences of the prey taxa are based on extensive ecological studies on various invertebrate groups carried out in the study area since 1960. (following:) non (non-agricultural/eurytopic including grassland), arable, wet (wetland/water), for (forest). (A) For sources of information on energy content based on ash-free dry mass (AFDM; 18–22), see also the bottom of the table.

Prey group/taxa	Habitat preference	Number of pellets in which a prey taxon was present		Total number of prey items			Individual dry mass (mg)	Energy content per individual (kJ)^A^	
**EARTHWORMS**									
Lumbricidae sp.	non	152		*c.* 4004			240	4.76 a	
**ORTHOPTERA**									
*Chorthippus* sp.	non	139		6066			40.6	0.91	
Metrioptera	non	138		5360			134.6	3.03	
*Tettigonia* sp.	non	96		760			1498.3	32.74	
*Gryllus campestris*	non	38		49			81.2	1.83	
*Gryllotalpa gryllotalpa*	non	8		11			90.0	2.02	
Orthoptera sp.	non	1		1			85.5	1.87	
**COLEOPTERA**									
*Silpha* sp.	non	108		545			26.0	0.60	
*Geotrupes* sp.	non	136		452			156.1	3.61	
*Pterostichus* sp.	non	124		366			54.2	1.25	
*Silpha obscura*	non	46		261			42.0	0.97	
*Carabus cancelatus*	non	117		227			125.8	2.91	
*Poecilus* sp.	arable	86		188			26.1	0.60	
*Hydrochara caraboides*	wet	62		138			29.0	3.61	
Coleoptera sp.	non	62		128			10.0	0.23	
*Zabrus tenebrioides*	arable	6		116			63.0	0.20	
*Amphimallon solstitialis*	non	20		92			225	5.21	
*Rhantus* sp.	wet	54		70			58.7	1.36	
Elateridae (larvae)	non	12		60			6.0	0.15	
*Selatosomus* sp.	arable	35		53			21.0	0.49	
*Ophonus* sp.	arable	37		48			8.5	0.20	
*Agriotes* sp.	arable	35		42			9.7	0.22 a	
*Cetonia* sp.	non	32		41			91.5	2.12	
*Amara sp.*	non	25		33			8.5	0.20	
Curculionidae	non	21		31			2.8	0.06	
*Agabus* sp.	wet	23		29			17.0	0.39 a	
*Calathus* sp.	arable	16		28			17.0	0.39	
*Phyllopertha* sp.	arable	6		22			17.4	0.40	
*Staphylinus* sp.	non	16		20			32.8	0.76	
*Bembidion* sp.	arable	12		17			1.2	0.03	
Histeridae	non	5		17			3.9	0.09	
*Otiorrhynchus* sp.	arable	16		17			37.3	0.86	
*Carabus auratus*	non	16		16			125.8	2.91	
*Carabus violaceus*	for	14		14			133.7	3.09	
*Trox* sp.	non	7		13			30.1	0.70	
*Colymbetes* sp.	arable	8		11			9.7	0.22	
*Necrophorus* sp.	non	10		11			265.9	6.15	
Chrysomelidae	non	8		10			7.2	0.17	
*Sitona* sp.	arable	10		10			4.7	0.11	
Elateridae	non	5		9			13.8	0.32	
*Helophorus sp.*	wet	9		9			0.3	0.01	
*Hydrous piceus*	wet	6		9			1293	16.62	
*Selatosomus latus*	arable	7		9			9.7	0.22	
Staphylinidae	non	8		9			1.8	0.04	
*Staphylinus cesareus*	non	7		8			10.0	0.23	
*Ceutorrhynchus* sp.	arable	6		7			0.8	0.02	
*Philonthus* sp.	arable	7		7			1.4	0.03	
*Cicindella* sp.	for	5		6			8.5	0.20	
Hydrophilidae	wet	3		6			1.0	0.02	
*Typhaeus typhoeus*	for	4		6			156.1	3.61	
*Anomala* sp.	arable	4		5			40.2	0.93	
*Carabus coriaceus*	for	4		5			1099	25.43	
Coleoptera (larvae)	non	4		5			6.0	0.15 a	
*Dorcus parallelipipedus*	for	5		5			251.6	5.81	
Buprestidae	non	3		4			5.4	0.12	
*Coccinella septempunctata*	non	4		4			13.7	0.32	
*Hydaticus* sp.	wet	3		4			13.7	0.32	
*Hydroporus* sp.	wet	3		4			13.7	0.32	
*Onthophagus* sp.	non	3		4			9.7	0.22	
*Phyllobius* sp.	arable	3		4			3.7	0.08	
*Apion* sp.	arable	3		3			0.5	0.01	
*Catops* sp.	non	1		3			1.1	0.03	
*Coreus marginatus*	non	3		3			37.2	0.86	
*Cryptopleurum* sp.	non	3		3			0.5	0.01	
*Cytilus sericeus*	non	3		3			5.2	0.12	
Dytiscidae (larvae)	wet	3		3			6.0	0.15	
*Dytiscus* (larvae)	wet	3		3			12.0	0.29	
*Hydrobius* sp.	wet	3		3			0.3	0.01	
*Liparus* sp.	for	3		3			119.1	2.76	
*Ontholestes* sp.	non	2		3			16.1	0.37	
*Oxytelus* sp.	arable	2		3			0.3	0.01	
*Prosternon tessellatum*	non	3		3			48.0	1.11	
Silphidae	non	1		3			145.9	3.38	
*Carabus* sp.	non	2		2			125.8	2.91	
*Cassida* sp.	non	2		2			12.0	0.28	
*Cercyon* sp.	arable	2		2			1.1	0.03	
*Dytiscus* sp.	wet	2		2			551	12.75	
*Hister* sp.	non	2		2			7.0	0.16	
*Lathrobium* sp.	non	2		2			1.6	0.04	
*Oulema melanopus*	arable	2		2			3.4	0.08	
*Propylaea 14-punctata*	non	2		2			3.2	0.07	
*Psylliodes chrysocephala*	arable	2		2			1.8	0.04	
Scarabaeidae	non	2		2			84.8	1.96	
*Spondylitis buprestoides*	for	2		2			125.8	2.91	
*Acilius* sp.	non	1		1			125.8	2.91 a	
*Anthicus* sp.	non	1		1			0.5	0.01	
*Aphodius* sp.	arable	1		1			6.7	0.16	
Carabidae	non	1		1			23.6	0.55	
*Chaetocnema* sp.	arable	1		1			0.9	0.02	
*Chalcophora mariana*	for	1		1			572.8	13.25	
Coccinellidae	non	1		1			4.4	0.10	
*Corticarina* sp.	non	1		1			0.2	0.005	
*Curculio* sp.	non	1		1			37.3	0.86	
Dytiscidae	wet	1		1			12.0	0.28	
*Glischrichilus* sp.	non	1		1			4.5	0.10	
*Graphoderes* sp.	arable	1		1			73.6	1.70	
*Hydraticus* sp.	wet	1		1			13.7	0.32	
Hydrophilidae (larvae)	wet	1		1			1.0	9.30	
*Hylobius* sp.	arable	1		1			37.3	0.86	
*Malachius* sp.	non	1		1			2.2	0.05	
*Necrodes* sp.	non	1		1			265.9	6.15	
Nitidulidae	arable	1		1			1.5	0.03	
*Oryctes nasicornis*	for	1		1			1145.6	26.51	
*Potamonectes* sp.	wet	1		1			8.5	0.20	
*Protaetia aeruginosa*	non	1		1			440	10.18	
Tenebrionidae	non	1		1			8.5	0.20	
*Xylodrepa* sp.	non	1		1			26.0		0.60
			
**OTHER INVERTEBRATES**									
*Lasius* sp.	non	81		316			0.6		0.014
Ichneumonidae	non	34		57			2.4		0.06
*Coreus* sp.	non	31		46			37.2		0.86
*Myrmica* sp.	non	25		46			1.2		0.03
*Forficula* sp.	non	21		40			11.7		0.27
*Mollusca*	non	32		33			200		3.56
Lepidoptera (larvae)	non	23		28			8.2		0.20
Diptera (larvae)	non	10		19			5.5		0.12
Araneae	non	11		13			4.3		0.10
*Nabis* sp.	non	9		11			2.0		0.05
*Formica* sp.	non	7		11			1.2		0.03
Odonata: Zygoptera	wet	6		10			137.6		3.13 b
Nematoda	non	4		9			3.0		0.06 c
*Aelia acuminata*	arable	8		8			14.3		0.33 a
Tenthredinidae	non	6		8			9.6		0.22
*Eurygaster maura*	arable	5		7			36.3		0.84
Insecta (larvae)	non	4		6			10.0		0.24
*Dolycoris* sp.	non	6		6			26.6		0.61
Pentatomidae	arable	4		6			26.2		6.02
*Viviparus viviparus*	wet	3		3			350		6.23 a
Heteroptera	non	3		3			2.0		0.05
Diplopoda	non	2		2			66.8		1.55
*Chartoscirta* sp.	wet	2		2			0.8		0.02
Apidae	non	2		2			19.8		0.45
Apoidea	non	2		2			19.8		0.45
*Bombus* sp.	non	2		2			50.7		1.15
*Panorpa sp.*	non	2		2			504.5		0.21 a
*Helix pomatia*	non	2		2			900.		16.02
*Orconestes limosus*	wet	1		1			5000		75.15 d
Diptera	non	1		1			2.0		0.04
*Graphosoma italica*	non	1		1			39.5		0.91
Lygaeidae	non	1		1			1.3		0.03
*Lygus sp.*	non	1		1			2.0		0.05
*Auchenorrhyncha* sp.	non	1		1			2.4		0.06
*Andrena* sp.	non	1		1			8.8		0.20
*Apis mellifera*	non	1		1			21.4		0.49
Eumenidae	non	1		1			4.8		0.11
*Camponotus* sp.	non	1		1			1.2		0.03
*Selenopsis* sp.	arable	1		1			1.2		0.03
*Vespula* sp.	non	1		1			25.7		0.59
Lepidoptera (eggs)	non	1		1			0.5		0.01
*Chrysopa* (larvae)	non	1		1			3.0		0.07
*Mollusca* (large)	non	1		1			1000		17.8
		
**FISH, REPTILES AND BIRDS**									
*Anguis fragilis*	for		92		95		6750	132.84 a	
Aves (small Passeriformes)	non		10		11		8200	191.22 a	
Pisces	wet		5		6		5000	110.75 a	
*Carassius carassius*	wet		2		2		5000	100.68 e	
*Lacerta* sp.	non		1		1		2700	53.14 a	
*Natrix natrix*	wet		1		1		24,300	478.22 a	
					
MAMMALS*									
*Microtus arvalis*	arable		80		81		6080	130.21 f	
*Talpa europaea*	non		38		39		19,520	429.97 f	
*Apodemus* sp.	non		8		8		6400	144.56 f	
*Arvicola amphibius*	wet		7		7		26,560	585.04 f	
*Myodes glareolus*	for		2		2		5440	117.74 f	
*Sorex* sp.	wet		1		2		1920	35.76 f	
*Microtus oeconomus*	wet		1		1		8320	183.26 f	

a Dolnik V.R., Dolnik T.V., Postnikov S.N. 1982. Caloric densities and metabolic efficiency coefficients of objects eaten by birds. In: Dolnik V.R. (Ed.) Time and energy budgets in free-living birds. Vol. 113: 143–153. Proceedings of Zoological Institute, Academy of Sciences of the USSR (in Russian).

b Caspers N. 1975. Kalorische Werte der dominierenden Invertebraten zweier Waldbäche des Naturparkes Kottenforst-Ville. Arch. Hydrobiol. 75, 4: 484–489.

c Prus T. 1970. Caloric value of animals as an element of bioenergetical investigations. Pol. Arch. Hydrobiol., 17, 183–199.

d Cummins K.W., Wuycheck J.K. 1971. Caloric Equivalents for Investigations in Ecological Energetics. Internationale Vereinigung für Theoretische und Angewandte Limnologie 18: 1–158. Stuttgart.

e P. Profus – unpubl data.

f Górecki A. 1965. Energy value of body in small mammals. Acta Theriologica 10, 23: 333–352.

* Note: It has been reported that a *c.* 7-day old nestling weighing 190 g (the oldest of the 4 nestlings in the nest) ingested mammalian prey items of the size of *Apodemus* sp. (P. Profus – unpubl.)

**Table 3:** All the statistically significant (*P* ≤ 0.05) results of the Spearman rank correlation coefficient (*r*_s_) testing the relationships between the various dietary indices determined for 165 pellets and landscape/habitat variables (i.e. area of individual land cover type expressed in ha or length of hydrographic networks expressed in m) measured at three spatial scales (1 km, 2.5 km and 5 km) around 52 White Stork nests in south-western Poland; *P*-values in bold meet the threshold of Bonferroni׳s correction at *α* ≤ 0.0036 (*k* = 14).

Land cover, habitat/ extent	N prey items		Total prey biome	Ind. prey mass	N taxa	Energy content per prey item		Total energy content	Biomass							%biomass							Biomass			%biomass	
									Earth	Cole	Orth	Other invert	Other verte	Mam		Earth	Cole	Orth	Other invert	Other verte	Mam		Invert	Verte		Invert	Verte
**Urban fabric n fabric**																											
1 km			-0.160						0.217		**-0.272**					**0.252**		**-0.328**		-0.192							
2.5 km																	0.202										
5 km				-0.170					**0.265**			0.193		-0.159		**0.268**			0.184		-0.174			-0.163		0.166	-0.164
																		
**Industrial, commercial and transport units**																											
2.5 km	0.170								0.196							0.173											
5 km	**0.278**			**-0.244**		-0.197			0.176	0.223		0.158											0.178				
																		
**Mine, dump and construction sites**																											
2.5 km																		-0.163									
5 km				-0.187					0.198			0.183	-0.161			0.205			0.166	-0.208							
																		
**Artificial non-agricultural vegetated areas**																											
1 km		-0.209			0.219			-0.208			**-0.256**		**-0.230**			0.228		**-0.259**		-0.217				-0.179		0.158	-0.160
2.5 km									**0.257**			0.158				**0.237**	-0.206			-0.160	-0.208		0.173	-0.164		0.218	-0.219
5 km	0.204			-0.186					0.213			0.216		-0.169		0.185			0.192	-0.187	-0.215		0.203	-0.179		**0.230**	**-0.229**
																					
**Arable land**																											
1 km									0.161																		
2.5 km		-0.184				-0.159			**0.264**		**-0.235**		-0.188	-0.194		**0.331**		**-0.241**						**-0.241**			
5 km	-0.197	**-0.324**			**-0.278**			**-0.353**			**-0.367**			-0.190		0.202	0.174	**-0.292**					**-0.234**	-0.219			
																				
**Grassland, pastures**																											
2.5 km																	-0.184										
5 km																	-0.171										
																			
**Heterogeneous agricultural areas**																											
1 km	-0.155			0.178		0.169												-0.164									
2.5 km				-0.185		-0.202			**0.252**				-0.216			**0.241**				**-0.254**				-0.193		0.203	-0.204
5 km	**0.274**			**-0.304**					0.187		0.195	0.174											0.181			0.176	-0.176
																					
**Forests**																											
1 km				0.203					**-0.312**				0.171			**-0.317**				0.203				0.206		-0.155	0.155
2.5 km		0.200			0.188			0.179	**-0.300**		**0.226**		0.178	0.214		**-0.354**		**0.250**						**0.248**			
5 km		0.213			0.225			0.197	-0.215		**0.252**		0.170	0.201		**-0.278**		**0.247**						**0.231**			
																	
**Shrub and/or herbaceous vegetation associations**																											
1 km					-0.200							-0.222	-0.178						-0.208	-0.188							
2.5 km									**-0.283**					0.179		**-0.250**	0.171				0.181						
5 km	0.164	0.173																		-0.173			0.196				-0.155
																				
**Inland wetland**																											
2.5 km				**0.254**		0.190			-0.174				0.174	0.192		-0.226								0.216		-0.170	0.170
5 km				**0.270**	**0.263**	**0.262**						-0.161							-0.187					0.157			
																					
**Inland water**																											
1 km	-0.208			**0.237**		0.189					-0.183		0.182					-0.174		0.222			-0.160			-0.159	0.159
2.5 km		0.180							0.209														0.194				
5 km									0.178				-0.176			0.165				-0.156							
																				
**Watercourses (length)**																											
1 km											0.191																
2.5 km											0.214						-0.219	0.179									
5 km											**0.237**						-0.200	0.211									
																		
**Shoreline of water bodies (length)**																											
1 km	-0.153	-0.160						-0.205			-0.196						0.184	-0.210									
2.5 km									0.179							0.182				-0.166							
5 km	0.169			-0.162		-0.166			0.196				-0.199			0.186				-0.206						0.166	-0.167
																	
**Water bodies and large rivers (surface area)**																											
1 km	**-0.291**	-0.200		0.187				-0.247			**-0.287**							**-0.246**					**-0.238**				
2.5 km									0.208							0.176				-0.155							
5 km							-0.153		0.212				-0.183			0.202				-0.174				-0.168		0.171	-0.172

**Table 4:** Comparison of landscape/habitat traits measured at three spatial scales for White Stork nests grouped into (A) pair productivity: low (1–2 fledglings; *n* = 21) and high (3–4 fledglings; *n* = 31), and (B) colonial breeding: solitary nests (*n* = 36) versus nests in aggregations (i.e. clumped distribution = more than one nest in an individual locality/village; *n* = 16); statistically significant results are shown in bold.

(A) **Pair productivity**						
Land cover type, hydrographic feature (unit)	1–2 fledglings		3–4 fledglings		Mann-Whitney test	
	Average	SE	Average	SE	Z	*P*-value
SPATIAL SCALE: 1 km						
Urban fabric (ha)	26	4	29	5	-0.55	0.585
Mine, dump and construction sites (ha)	0.0	0.0	3.5	2.9	-1.18	0.240
Artificial non-agricultural vegetated areas (ha)	0.00	0.00	0.26	0.26	-0.82	0.410
Arable land (ha)	197	18	183	16	0.49	0.621
Grassland, pasture (ha)	39	10	34	7	0.23	0.821
Heterogeneous agricultural areas (ha)	4.1	2.2	9.9	3.5	-1.04	0.299
Forests (ha)	44.6	12.9	52.9	12.0	-0.84	0.398
Shrub and/or herbaceous vegetation associations (ha)	3.2	1.8	0.1	0.1	1.51	0.130
Inland wetlands (ha)	0.0	0.0	0.0	0.0	–	–
Inland waters (ha)	0.9	0.8	1.5	0.9	-0.37	0.712
**Watercourses** (m)	**11,554**	**1260**	**8712**	**879**	**2.25**	**0.025**
Shoreline of water bodies (m)	1629	528	2242	438	-1.78	0.075
Inland waters and large rivers (ha)	2.5	1.2	4.0	1.3	-1.69	0.091

SPATIAL SCALE: 2.5 km						
Urban fabric (ha)	54	11	77	16	-0.85	0.396
Industrial, commercial and transport units (ha)	2.5	1.9	4.9	2.9	0.53	0.596
Mine, dump and construction sites (ha)	5	4	8	6	-0.34	0.737
Artificial non-agricultural vegetated areas (ha)	19	6	11	4	1.16	0.248
Arable land (ha)	905	90	922	77	-0.08	0.933
Grassland, pasture (ha)	286	41	224	36	1.32	0.185
Heterogeneous agricultural areas (ha)	94	14	84	12	0.52	0.601
Forests (ha)	494	87	561	81	-0.33	0.744
Shrub and/or herbaceous vegetation associations (ha)	21	8	5	2	1.50	0.133
Inland wetlands (ha)	0.4	0.4	0.5	0.5	0.25	0.801
Inland waters (ha)	50	9	33	7	1.51	0.131
Watercourses (m)	58,670	5210	47,681	3724	1.87	0.061
Shoreline of water bodies (m)	17,508	2685	15,153	1870	0.62	0.532
Inland waters and large rivers (ha)	64	11	47	8	0.96	0.337

SPATIAL SCALE: 5 km						
Urban fabric (ha)	338	45	337	36	-0.07	0.941
Industrial, commercial and transport units (ha)	166	38	94	26	1.20	0.229
Mine, dump and construction sites (ha)	46	11	38	10	0.79	0.428
Artificial non-agricultural vegetated areas (ha)	96	28	66	20	0.79	0.428
Arable land (ha)	2585	273	3030	246	-0.81	0.417
Grassland, pasture (ha)	1036	117	767	93	1.71	0.088
Heterogeneous agricultural areas (ha)	290	27	274	23	0.55	0.582
Forests (ha)	2880	315	2905	236	-0.33	0.744
Shrub and/or herbaceous vegetation associations (ha)	119	18	81	14	1.73	0.085
Inland wetlands (ha)	12	7	9	6	0.28	0.780
Inland waters (ha)	158	23	125	18	1.13	0.259
Watercourses (m)	175,753	12,683	157,049	8728	1.41	0.159
Shoreline of water bodies (m)	63,580	6928	54,090	5491	1.04	0.301
Inland waters and large rivers (ha)	231	33	179	25	1.13	0.259

**(B) Colonial breeding**						
Land cover type, hydrographic feature (unit)	Solitary		Aggregation		Mann-Whitney test	
	Average	SE	Average	SE	*Z*	*P*-value
SPATIAL SCALE: 1 km						
Urban fabric (ha)	27	5	31	2	-1.50	0.133
Mine, dump and construction sites (ha)	3.0	2.5	0.0	0.0	0.95	0.341
Artificial non-agricultural vegetated areas (ha)	0.2	0.2	0.0	0.0	0.67	0.505
**Arable land** (ha)	**161**	**15**	**251**	**12**	**-3.33**	**0.001**
Grassland, pasture (ha)	40	7	28	10	0.37	0.711
**Heterogeneous agricultural areas** (ha)	**10.8**	**3.2**	**0.3**	**0.3**	**2.06**	**0.039**
**Forests** (ha)	**70**	**11**	**5**	**3**	**4.41**	**0.000**
Shrub and/or herbaceous vegetation associations (ha)	1.9	1.1	0.0	0.0	1.37	0.170
Inland wetlands (ha)	0.0	0.0	0.0	0.0	–	–
Inland waters (ha)	1.8	0.9	0.0	0.0	1.72	0.086
**Watercourses** (m)	**8015**	**655**	**14,010**	**1511**	**-3.83**	**0.000**
Shoreline of water bodies (m)	2479	459	904	178	1.09	0.275
Inland waters and large rivers (ha)	4.5	1.3	0.9	0.1	0.93	0.351
SPATIAL SCALE: 2.5 km						
**Urban fabric** (ha)	**80**	**14**	**40**	**10**	**2.22**	**0.026**
Industrial, commercial and transport units (ha)	4.9	2.7	1.6	0.7	-1.90	0.057
Mine, dump and construction sites (ha)	9.7	5.3	0.0	0.0	0.95	0.341
**Artificial non-agricultural vegetated areas** (ha)	**3.0**	**1.6**	**39.7**	**5.9**	**-4.12**	**0.000**
**Arable land** (ha)	**827**	**75**	**1114**	**58**	**-2.54**	**0.011**
**Grassland, pasture** (ha)	**212**	**34**	**332**	**37**	**-2.52**	**0.012**
**Heterogeneous agricultural areas** (ha)	**70**	**10**	**129**	**13**	**-2.87**	**0.004**
**Forests** (ha)	**684**	**73**	**197**	**18**	**3.69**	**0.000**
Shrub and/or herbaceous vegetation associations (ha)	15.5	4.7	3.4	3.1	1.41	0.159
Inland wetlands (ha)	0.7	0.5	0.0	0.0	0.32	0.751
**Inland waters** (ha)	**25**	**6**	**75**	**8**	**-3.87**	**0.000**
**Watercourses** (m)	**43,426**	**3048**	**71,678**	**4686**	**-3.99**	**0.000**
**Shoreline of water bodies** (ha)	**13,480**	**1959**	**22,008**	**1719**	**-3.19**	**0.001**
**Inland waters and large rivers** (ha)	**36**	**7**	**93**	**9**	**-3.61**	**0.000**
SPATIAL SCALE: 5 km						
**Urban fabric**	**288**	**34**	**449**	**39**	**-3.03**	**0.002**
**Industrial, commercial and transport units** (ha)	**66**	**21**	**252**	**37**	**-3.39**	**0.001**
**Mine, dump and construction sites** (ha)	**30**	**10**	**68**	**9**	**-3.35**	**0.001**
**Artificial non-agricultural vegetated areas** (ha)	**22**	**10**	**204**	**30**	**-3.65**	**0.000**
Arable land (ha)	3011	236	2487	263	1.19	0.234
**Grassland, pasture** (ha)	**652**	**75**	**1378**	**84**	**-4.46**	**0.000**
**Heterogeneous agricultural areas** (ha)	**252**	**20**	**343**	**30**	**-2.78**	**0.006**
**Forests** (ha)	**3208**	**237**	**2189**	**213**	**2.48**	**0.013**
**Shrub and/or herbaceous vegetation associations** (ha)	**84**	**15**	**123**	**11**	**-2.26**	**0.024**
Inland wetlands (ha)	14,8	6,4	0,0	0,0	0.95	0.341
**Inland waters** (ha)	**96**	**14**	**233**	**19**	**-4.52**	**0.000**
**Watercourses** (m)	**150,451**	**9153**	**196,445**	**7582**	**-3.07**	**0.002**
**Shoreline of water bodies** (m)	**45,715**	**4452**	**85,391**	**5370**	**-4.40**	**0.000**
**Inland waters and large rivers** (m)	**134**	**17**	**349**	**29**	**-4.62**	**0.000**

**Table 5:** Comparison of dietary indices/variables of breeding White Storks (A) among nests with low productivity (1–2 fledglings; *n* = 66 pellets) and high productivity (3–4 fledglings; *n* = 99 pellets) pairs and (B) among solitary nests (*n* = 125 pellets) and nests in an aggregation (i.e. more than one nest in an individual locality/village; *n* = 40 pellets); statistically significant results are shown in bold. Note: Thirty-two pellets were collected from 12 nests in Kłopot, a village supporting one of the largest White Stork colonies in Poland, see [1].

**(A) Pair productivity**						
Dietary index/variable (unit)	1–2 fledglings		3–4 fledglings		Mann-Whitney test	
	Average	SE	Average	SE	Z	*P*-value
Biomass of earthworms (mg d.w.)	5379	882	5997	782	-0.43	0.670
**Biomass of Coleoptera (mg d.w.)**	**3773**	**1275**	**1811**	**157**	**1.99**	**0.046**
**Biomass of Orthoptera (mg d.w.)**	**18,349**	**2215**	**9074**	**1402**	**4.21**	**0.000**
Biomass of other invertebrates (mg d.w.)	121.3	22.6	143.8	55.4	1.78	0.075
Biomass of other vertebrates (mg d.w.)	5967	611	5103	510	1.35	0.177
Biomass of mammals (mg d.w.)	7719	1115	9361	1267	-0.12	0.904
%biomass of earthworms	13.9	2.3	20.9	2.6	-1.77	0.077
%biomass of Coleoptera	8.3	1.4	6.9	0.6	-0.05	0.963
**%biomass of Orthoptera**	**39.2**	**3.3**	**24.2**	**2.3**	**3.55**	**0.000**
%biomass of other invertebrates	0.3	0.1	0.5	0.1	0.70	0.482
%biomass of other vertebrates	17.0	2.1	17.5	1.8	-0.29	0.775
%biomass of mammals	21.4	2.8	30.1	3.1	-1.44	0.149
**Number of prey items**	**149.0**	**12.1**	**108.2**	**10.2**	**3.22**	**0.001**
**Total biomass of prey** (mg d.w.)	**41,307**	**2620**	**31,489**	**2067**	**3.26**	**0.001**
Individual prey mass (mg d.w.)	397.0	40.7	481.8	45.6	-0.60	0.545
**Number of prey taxa**	**16.3**	**0.6**	**14.5**	**0.5**	**2.14**	**0.032**
**Biomass of all invertebrates** (mg d.w.)	**27,622**	**2610**	**17,025**	**1803**	**4.00**	**0.000**
Biomass of all vertebrates (mg d.w.)	13,685	1308	14,464	1353	0.30	0.761
%biomass of all invertebrates	38.3	3.3	47.6	3.1	-1.94	0.052
%biomass of all vertebrates	61.7	3.3	52.4	3.1	1.94	0.052
Energy content per 1 prey item (kJ)	8.1	0.8	9.8	1.0	-0.189	0.850
**Total energy content** (kJ)	**840**	**53**	**663**	**44**	**2.97**	**0.003**

**(B) Colonial breeding**						
Dietary index/variable (unit)	Solitary		Aggregation		Mann-Whitney test	
	Average	SE	Average	SE	Z	*P*-value
**Biomass of earthworms (mg d.w.)**	**4311**	**557**	**10,245**	**1478**	**-3.84**	**0.000**
Biomass of Coleoptera (mg d.w.)	2876	685	1720	188	0.68	0.494
Biomass of Orthoptera (mg d.w.)	12,260	1493	14,418	2380	-1.66	0.096
Biomass of other invertebrates (mg d.w.)	103	18	234	131	-1.27	0.205
Biomass of other vertebrates (mg d.w.)	5647	407	4828	1002	1.61	0.108
**Biomass of mammals** (mg d.w.)	**9088**	**900**	**7504**	**2317**	**2.08**	**0.038**
**%biomass of earthworms**	**14.5**	**1.9**	**28.9**	**4.1**	**-3.53**	**0.000**
**%biomass of Coleoptera**	**8.20**	**0.85**	**5.19**	**0.70**	**1.97**	**0.049**
%biomass of Orthoptera	29.6	2.3	32.6	3.7	-0.91	0.364
%biomass of other invertebrates	0.37	0.06	0.46	0.18	-0.57	0.566
%biomass of other vertebrates	18.04	1.47	15.02	3.22	1.70	0.090
**%biomass of mammals**	**29.34**	**2.49**	**17.84**	**4.35**	**2.71**	**0.007**
**Number of prey items**	**114.1**	**8.8**	**156.8**	**17.2**	**-2.62**	**0.009**
Total biomass of prey (mg d.w.)	34,286	1960	38,950	3054	-1.54	0.125
**Individual prey mass** (mg d.w.)	**479**	**39**	**350**	**45**	**2.02**	**0.043**
Number of prey taxa	15.0	0.5	15.9	0.7	-0.3	0.798
**Biomass of all invertebrates** (mg d.w.)	**19,550**	**1776**	**26,619**	**3079**	**-2.67**	**0.008**
**Biomass of all vertebrates** (mg d.w.)	**14,735**	**1026**	**12,332**	**2353**	**2.03**	**0.042**
**%biomass of all vertebrates**	**52.6**	**2.6**	**67.1**	**4.7**	**-2.69**	**0.007**
**%biomass of all invertebrates**	**47.4**	**2.6**	**32.9**	**4.7**	**2.69**	**0.007**
Energy content per 1 prey item (kJ)	9.7	0.8	7.4	1.0	1.46	0.145
Total energy content (kJ)	706	40	823	66	0.09	0.089

The average mass of one individual prey item calculated across all identified prey (*n* = 20 561; Table 2) was 286 mg (95% C.I. = 269–302 mg), while the average mass of one individual prey item per pellet (n = 165) and per nest (*n* = 52) was 445 mg (95% C.I. = 384–510 mg) and 399 mg (95% C.I. = 335–462 mg), respectively. The total biomass (dry mass) of all prey consumed was 5869 g (Table 2).

### 1.1 Data on overall diet composition and prey composition in White Storks

The most numerous prey group both by number and biomass was Orthoptera (59.5% and 35.6%, respectively). The following ranking for invertebrate prey items in descending order of their quantitative contribution to the diet was: earthworms (19.5% by number and 16.4% by biomass), Coleoptera (16.2% and 7.3%) and other invertebrates (3.5% and 0.4%). Small mammals and other vertebrates (i.e. fish, reptiles and birds) constituted only 0.7% and 0.6% of all prey items consumed, but the contribution of their biomass was disproportionately high at 24.8% and 15.2%, respectively (Table 2).

With regard to the functional division of prey, the diet of White Storks was based primarily on ‘eurytopic prey’ (embracing taxa from grassland and a variety of non-cropped habitats), the biomass contribution of which in the diet was disproportionately (3–4–fold) higher than the percentage of available corresponding habitats (Fig. 2). Similarly, the prey from water/wetland sites prevailed over the availability of corresponding habitats (Fig. 2). The opposite pattern characterized prey taxa from arable habitats and forests, the contribution of which was lower that the availability of the corresponding habitats (Fig. 2).

On average (confidence interval = C.I.) per 1 nest (*n* = 52), invertebrate prey and vertebrate prey respectively made up 58% (95% C.I. = 52–64%) and 42% (95% C.I. = 36–48%) of the biomass consumed.

The total energy content per pellet (calculated by summing the energy content of all individual prey items across one specific prey group) was the most strongly correlated with the biomass of Orthoptera (Pearson *r* = 0.801, *P* < 0.0001), thereafter with that of mammals (*r* = 0.361, *P* < 0.0001), other vertebrates (*r* = 0.234, *P* = 0.002), earthworms (*r* = 0.214, *P* = 0.006) and other invertebrates (*r* = 0.181, *P* = 0.020), but not with the biomass of Coleoptera (*r* = 0.024, *P* = 0.756) (see also Table 3).

Across the 52 nests analysed the diet was based primarily on prey items attributed to the grassland/non-cropped habitat category collectively referred to as ‘eurytopic prey’, and their biomass contribution to the diet was significantly (indexed via the *t*-test) – 3–4–fold – and disproportionately higher than the percentage of available corresponding habitats at each of the three distances (1 km, 2.5 km and 5 km) around the nests (Fig. 2). Similarly, the contribution of prey items from water/wetland sites prevailed significantly – 2–5–fold – over the availability of corresponding habitats (Fig. 2). The opposite pattern characterized prey taxa from arable habitats, the contribution of which was markedly – 3–5–fold – lower than the availability of corresponding habitats. Lastly, the contribution of the prey category attributed to forest habitats was similar to that of the availability of these habitats measured at the distance of 1 km around nests, but was significantly – 2–3–fold – lower than the availability of forests at the other two distances (Fig. 2).

MANOVA revealed significant differences in dietary composition in terms of the biomass of the six major prey groups (earthworms, Orthoptera, Coleoptera and other invertebrates, other vertebrates and mammals) identified in the 52 nests (MANOVA, Wilks׳s Lambda, *λ*_306,653_= 0.007, *P* < 0.0001). However, further *post-hoc* analysis (yielding a matrix with 1 326 comparisons between pairs of nests for each individual prey group) confirmed that the contribution of two prey groups – small mammals and other invertebrates – did not differ between any pairs of nests, indicating similar exploitation of these prey resources across the entire landscape in which our White Stork population foraged. The biomass contribution of the other four prey groups varied between different nests with variable magnitude. So, the negligible variations between nests observed in the case of biomasses of Coleoptera, other vertebrates and earthworms, which varies significantly merely between 3, 5 and 12 pairs of nests, respectively. The most variable contribution was that of the biomass of Orthoptera, which varied between 55 pairs (from all 1326 possible comparisons) of nests.

The variations in biomass of the major prey groups and individual prey mass for different contributions of grassland and arable land measured within three spatial scales around the nests are visualized in Fig. 3.

### 1.2 Data on reproductive output in relation to colonial breeding and habitat variation

On average, the number of fledglings in solitary nests (*n* = 36) was nearly 14% higher than in nests with a clumped distribution (*n* = 16): 2.69 (95% C.I. = 2.45–2.93) vs 2.37 (95% C.I. = 2.04–2.70), respectively; however, this difference was not significant (Mann-Whitney test, Z = 1.46, *P* = 0.145), presumably because of the small sample size. This result may be explained in part by the fact that there is significantly less %arable land (within 1 km and 2.5 km) around solitary nests than in the vicinity of clumped nests; in contrast, %grassland was higher and there were more aquatic habitats (mostly within 2.5 km and 5 km) around the clumped nests (Table 4).

MANOVA did not show any significant effect (*P* ≤ 0.251) of the percentage distribution of the eleven land cover types (Fig. 4) or four major habitat categories used for prey classification (i.e. certain land cover types combined; Fig. 2) influencing the number of nestlings in the nest. However, inspection of the distribution of the percentage of the four major land cover types around nests with different numbers of fledglings yielded a single, clear pattern: this tallied only in part with our initial prediction. So, %arable land consistently decreased with the number of fledglings across all three distances (1, 2.5 and 5 km) around nests, whereas %forest exhibited the opposite pattern, this percentage increasing along with the number of fledglings (Fig. 4). Furthermore, we found that in principle, there were no differences between the nests of low productivity pairs (1–2 fledglings) and those of high productivity pairs (3–4 fledglings) in any of the landscape traits analysed (Table 4).

We found marginally significant differences (both at *P* = 0.08) for two dietary indices, the biomasses of five major prey groups and number of prey taxa, between nests with different numbers of fledglings (Table 5, Fig. 4).

White Storks from the nests of low productivity pairs (i.e. with 1–2 fledglings) consumed a significantly (up to two-fold) higher biomass of Coleoptera, Orthoptera and all invertebrates, which also translated into a higher total biomass and a higher total energy content compared to the diet of high-productivity pairs (Table 5).

## 2 Experimental design, materials and methods

As the majority of nestling mortality occurs during the first 20 days after hatching [2], [3], [4], we considered the number of fledglings present in a nest at the time of ringing to be a proxy of the productivity of a breeding pair of White Storks (hereafter ‘reproductive output’).

The estimates of earthworm biomass were based on a soil mass of 192 mg per 1000 chaetae (for more details see also Orłowski et al. [5]). For each pellet, the number of prey items representing each invertebrate taxon was established from the numbers of fragments of chitinous body parts (according to [6], [7]). Here, we added two new eco-morphological characteristics for each individual prey item: (1) energy content (expressed in kJ) and (2) functional division in terms of habitat preferences (Table 2). The energy content (ash-free dry mass, AFDM) in prey items of White Storks (see Table 2) followed [8], [9], where previous data for specific (or related) prey taxa were used (after [10], [11], [12], [13], [14]).

We applied the functional division of the individual prey species/taxa in terms of their habitat preferences (see Table 2), in part basing this classification on our previous detailed per-taxa habitat assignment [15]. Specifically, we classified the individual prey species/taxa into four major habitat categories, taking into account their relationship with the landscape and agricultural activities as the habitat of their development and their association with crop or non-crop habitats [15]. The habitat preferences of prey taxa were based on extensive ecological studies of various invertebrate groups in agricultural regions of south-western Poland after 1960 ([16], [17], [18], [19], [20], [21]; summarized in [15]). This yielded four groups of prey from i) non-agricultural/marginal habitats including grassland, ii) crop fields/arable land, iii) wetland and aquatic habitats, and iv) forest and woodland habitats (Table 1).

The habitat preferences of prey taxa were based on extensive ecological studies of various invertebrate groups in agricultural regions of south-western Poland after 1960 ([16], [17], [18], [19], [20], [21]; summarized in [15]). This yielded four groups of prey from i) non-agricultural/marginal habitats including grassland, ii) crop fields/arable land, iii) wetland and aquatic habitats, and iv) forest and woodland habitats (Table 1).

### 2.1 Statistical analysis

The aim of our analysis of data in Fig. 2 was to assess whether the percentage distribution of the biomasses of four functional prey groups representing taxa from major habitat categories (arable; eurytopic = grassland/non-cropped; forest; and waters/wetland) was utilized in proportion to the availability of the corresponding habitats measured at three different distances (1 km, 2.5 km and 5 km) around the same 52 White Stork nests. The corresponding background of available habitats is a synthetic measure combining land cover classes with a similar structure: arable (Arable land + Heterogeneous agricultural areas), grassland/non-cropped (Urban fabric + Industrial, commercial and transport units + Mine, dump and construction sites + Grassland, pasture + Shrub and/or herbaceous vegetation associations), forest (Forests + Artificial non-agricultural vegetated areas) and water/wetland (Inland wetlands + Inland waters and large rivers) (Table 1; Fig. 1; see also [1]). The percentage distribution of individual prey groups vs available habitat background (Fig. 2) was compared with using the *t*-test for dependent samples.

Finally, since previous findings on behavioural limitations resulted from colonial breeding leading to decreased reproductive output in White Storks, we compared using the Mann-Whitney test, the landscape traits and dietary indices between nests of low productivity pairs (1–2 fledglings; *n* = 21 nests) and nests of high productivity pairs (3–4 fledglings; *n* = 31 nests); and (2) between solitary nests (*n* = 36) and nests in an aggregation (*n* = 16; Table 4; Table 5). However, results of the latter analysis due to non-random sampling design (i.e. the true density of ׳solitary׳ and ׳colonial׳ nests is unknown) should be treated with caution.
