# Supplementary material for: Data exploration on diet, and composition, energy value and functional division of prey items ingested by White Storks Ciconia ciconia in south-western Poland: Dietary variation due to land cover, reproductive output and colonial breeding
Source: Data Brief. 2018 Oct 24;21:1186–203. doi: 10.1016/j.dib.2018.10.064 (PMC6230975; doi:10.1016/j.dib.2018.10.064)
Supplement: Supplementary file 1 — Supplementary material [file mmc1.doc]

**Conflict of interest**

The authors declared they do not have anything to disclose regarding conflict of interest with respect to this manuscript.
